# Ecofriendly Composite as a Promising Material for Highly-Performance Uranium Recovery from Different Solutions

**DOI:** 10.3390/toxics10090490

**Published:** 2022-08-24

**Authors:** Mohammed F. Hamza, Hanaa A. Abu Khoziem, Mahmoud S. Khalafalla, Walid M. Abdellah, Doaa I. Zaki, Khalid Althumayri, Yuezhou Wei

**Affiliations:** 1 School of Nuclear Science and Technology, University of South China, Hengyang 421001, China; 2 Nuclear Materials Authority, POB 530, El-Maadi, Cairo 11728, Egypt; 3 Department of Chemistry, College of Science, Taibah University, Al-Madinah Al-Munawarah 30002, Saudi Arabia; 4 School of Nuclear Science and Engineering, Shanghai Jiao Tong University, Shanghai 200240, China

**Keywords:** uranium, metal decontamination, hydrogel, uptake kinetics, sorption isotherms, recovery of heavy metal

## Abstract

The development of new materials based on biopolymers (as renewable resources) is substantial for environmental challenges in the heavy metal and radionuclide ions removal contaminations. Functionalization of chitosan with sulfonic groups was achieved for improving the uranium sorption, not only from slightly acidic leachate, but also for the underground water. The prepared hydrogel based on chitosan was characterized by series of analysis tools for structure elucidation as FTIR spectroscopy, textural properties using nitrogen adsorption method, pH_PZC_ (by pH-drift method), thermogravimetric analysis (TGA), SEM, and SEM-EDX analyses. The sorption was performed toward uranium (VI) ions for adjustment of sorption performances. The optimum sorption was performed at pH 4 (prior to the precipitation pH). The total sorption was achieved within 25 min (relatively fast kinetics) and was fitted by pseudo-first order rate equation (PFORE) and resistance to intraparticle diffusion equation (RIDE). The maximum sorption capacity was around 1.5 mmol U g^−1^. The sorption isotherms were fitted by Langmuir and Sips equations. Desorption was achieved using 0.3 M HCl solution and the complete desorption was performed in around 15 min of contact. The sorption desorption cycles are relatively stable during 5 cycles with limit decreasing in sorption and desorption properties (around 3 ± 0.2% and 99.8 ± 0.1%, respectively). The sorbent was used for removal of U from acid leachate solution in mining area. The sorbent showed a highly performance for U(VI) removal, which was considered as a tool material for radionuclides removing from aquatic medium.

## 1. Introduction

The hazardous metal removal and the valuable metal recovery are challenging techniques for water purification and metal valorization. Different processes were used either in leaching of metal ions from ores, waste materials or extraction processes from solution. The green leaching using Humic acid (HA), as well as mineral acids, were used for removing of uranium from crude and spent materials [1,2,3,4]. For this purpose, several techniques were used including solvent extraction, metal precipitation, and membrane-based techniques, which faced limited use on the concentration level of metal ions and the solution complexity. Sorption is the more specifically used in the low concentration of metal ions. Although there is a large variety of commercial resins, there is a demand for developing special types of sorbents (nano particles size or hydrogel) for the removal of heavy metal ions as well as recovering of valuable/economic metals.

A wide variety of functionalized sorbents were identified for recovering valuable metals and removing hazardous elements from the aquatic medium through high sorption capacity and selectivity, in addition to uses in the diluted solution treatments as wastewater or groundwater treatment. These include (a) combination of hydrogels (i.e., alginate and chitosan [5]), (b) Sulfonic functionalized materials, yeast cells [6,7,8,9,10], (c) Amidoxime groups on synthetic sorbents [11,12,13,14,15] (d) Quaternary ammonium-based sorbents [16,17], (e) bio-based composites [18,19,20,21,22], metal-organic framework [23], or (f) Iminodiacetic acid [24].

Industrial effluents and wastewater treatment through heavy and radionuclide removal is a crucial prospective and main target of researchers. The development of the high-tech industries led to increasing the various pollutants, (heavy elements as lead and cadmium, and the increasing demand of nuclear power increasing the pollution of uranium and thorium, which have had a very negative impact on both environment and human beings). These elements can enter the human body directly through water and food [10,25,26,27,28]. The importance of this point is due to the increased necessity from several governments for community protection [29]. Discharging of the mining effluents causes several health problems. For making valorization of such process, by recycling valuable and rare metals that are included in such solution. Several methods were used for removing of contaminants such as solvent extraction [30], precipitation [31,32], membrane separation and electrolytic techniques [33]. This faces a problem in technical and economic uses, especially for diluted solution. Ion exchangers and chelating sorbents are a suitable tools metal recovery, even in the low metal concentration [5,34,35,36,37,38].

In previous decades, nanoparticles modified sorbents received a prominent attention due to the fast kinetic sorption and highly sorption capacities. The natural biobased materials as alginate, chitosan, algal biomass, or agriculture wastes can be designed as micro or nano materials [39,40,41]. These materials have several functional groups as hydroxyls and amines, which gives reasons for the hydrophilic properties and are ready for further modification through functionalization with other groups to enhance the capacity and improve kinetics.

Chitosan substrate has a good property toward sorption of metal ions. As functionalization was achieved, the sorption properties, as well as selectivity, were changed depending on the type of sorption (as electrostatic attraction or ionic exchange (at acidic pH value) or chelation properties (at slightly acidic or neutral pH values)) [14,42,43,44,45]. The grafted groups improve the physical properties of the sorbent through sorption in a broad pH value. In parallel application with metal removal on some prepared sulfonated sorbents, complementary tests were used for the antimicrobial effect. Most of these composite derivatives have been reported as a reactive to the antimicrobial activity [46].

This study compares the sorption properties of U(VI) on functionalized sulfonic groups (derived from 2-Acrylamido-2-methylpropane sulfonic acid (AMPS)) as well as the amine groups (from chitosan and N,N’-Methylenebisacrylamide (MBA; the crosslinker)) of the functionalized composites hydrogel. The prepared sorbent was characterized by textural properties (BET), FTIR spectroscopy, and morphological characterizations (SEM, and SEM-EDX), thermogravimetric analysis (TGA). The sorption properties towards U(VI) were investigated through sorption tests including pH effect, uptake kinetics, sorption isotherms, selectivity (sorption from multi-component solutions), metal desorption and sorbent recycling. The material was investigated for feasibility evaluation through industrial applications, with the composite being tested on real solution bearing complex metal ions.

## 2. Materials and Methods

### 2.1. Materials

The chemicals used in this study are of analytical grade. N,N-Methylenebis(acrylamide) (MBA; 99%), potassium persulfate (>99%), chitosan (Medium molecular weight; 75–85% of AD, acetylation degree), sodium hydroxide (≥98%), and Bromoform (99%) contains 60–120 ppm 2-methyl-2-butene as stabilizer, were supplied from Sigma Aldrich (Frankfurter Str. 250, 64293-Darmstadt-Germany). 2-Acrylamido-2-methylpropane sulfonic acid (AMPS) was supplied from SHANDONG ZHI SHANG CHEMICAL Co., Ltd.-China; Hisense Intelligence Vally, High-tech Zone, Jinan, Shandong.

### 2.2. Synthesis of Sorbent

#### 2.2.1. Synthesis of the Reference Material (Chitosan Crosslinked)

**Scheme 1:**
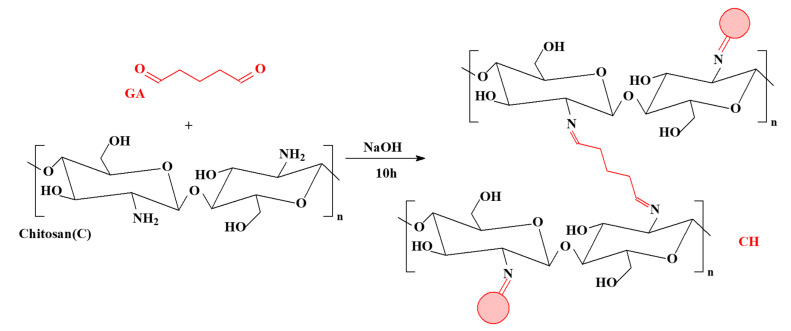
Synthesis of the CH sorbent (as a reference material).

Dissolving of 2 g of chitosan particles in 30 mL of 7% (*v*/*v*) acetic acid solution was performed in a three nicked flask (equipped with condenser, agitator, and thermostat). Addition of 5 mL (15% *v*/*v*) glutaraldehyde solution dropwise with vigorous stirring. The produced precipitation was continued stirring (with velocity 190 rpm) for 5 h at 75 °C before filtered and washed with acetone and water to remove the unreacted materials and dried at 60 °C for 10 h to yield CH, as shown in Scheme 1.

#### 2.2.2. Synthesis of the Functionalized Chitosan Composite

**Scheme 2:**
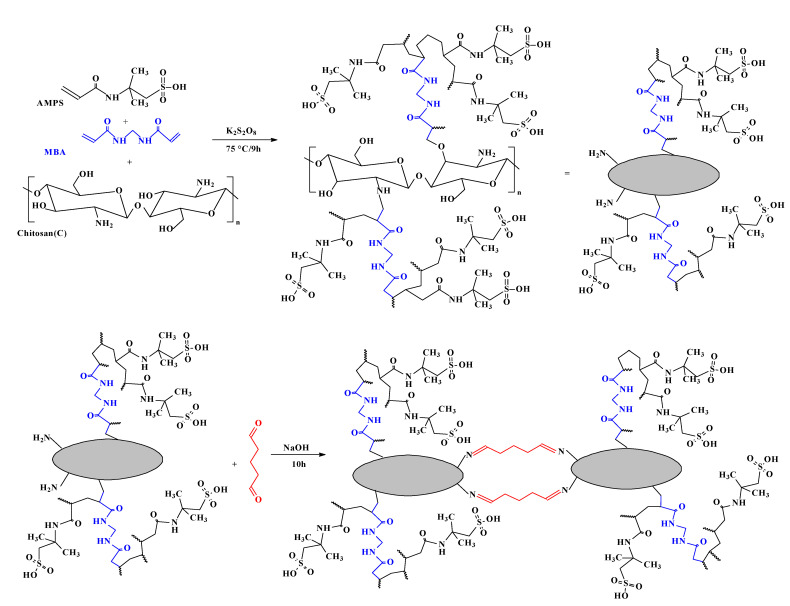
Synthesis route of the sulfonic hydrogel (CH-S).

Two grams of chitosan and 0.2 g of potassium persulfate were dissolved in 7% (*v*/*v*) acetic acid solution (50 mL) in three nicked flask. There as an addition of 2 g of AMPS and 0.1 g of MBA (as a crosslinker) to the solution with vigorous stirring (190 rpm) till dissolve. The flask was equipped with a spiral condenser and heated at 75 °C for 9 h under the vigorous stirring conditions. After cooling, the mixture was poured to a 500 mL solution of NaOH (0.5% (*w*/*v*)) contains 15% *v*/*v* glutaraldehyde solution (for enhancing the stability through further crosslinking effect) for 10 h at room temperature (21 ± 2 °C) with gentle stirring (95 rpm). The precipitated hydrogel was filtered and washed several times with deionized water and acetone then dried at 60 °C for 10 h to yield CH-S as shown in Scheme 2.

### 2.3. Characterization of Materials

The produced sorbents (CH and CH-S) were subjected to grind and sieving. The sorbents that used in the experiments were obtained with scale up to 10 µm. The (C, S, N and H composition) was measured using elemental analysis through element analyzer; CHNOS; Vario ELIII, Elementar-Analyzer system; GmbH, Sonaustraβe-20354 Hamburg, Germany. Chemical analysis and morphological studies were performed by the scanning electron microscope joint with the energy dispersive X-ray analyzer (SEM and EDX, respectively); XL30-ESEM; Philips, Thermo Fisher Scientific, Hillsboro, OR, USA. FTIR spectroscopy (Fourier-transform infrared) were used from the Mobile IR-Portable; Bruker Optics, Billerica, MA, USA. The textural properties of the synthesized material were carried out through the high-speed surface area analyzer using Nova-e Series, Model-25, Quantachrome, Kingsville, TX, USA. The thermal analysis for material degradation was carried out by the TGA thermogravimetric analyzer; N5320011-Perkin Elmer, Villebon-sur Yvette, France), using nitrogen atmosphere as medium for the analysis, with 10 °C min^−1^ as a temperature ramp. The pH_PZC_ (zero-charge) was measured by the pH-drift method; a 100 mg of the prepared sorbents (both CH and CH-S) were mixed with 50 mL of series of prepared solution (11 prepared samples in closed bottle) with concentration of 0.1 M NaCl at fixed pH_0_ (initial pH), that ranged between 1 and 11. After stirring for around 48 h, the pH_f_ (final pH) was measured (using pH meter with the specification described below) and the difference in pH values of the initial and final were determined, in which the pH_PZC_ was equal to pH_0_ = pH_f_. The pH of the solution was established through pH/ionometer, S220 Seven compact, Mettler Toledo-China. The collected solution (samples after pH effect, sorption isotherms, uptake kinetics, desorption, selectivity and after treatment with real samples) was firstly filtrated using micromembrane (1.2 µm) before measuring the metal contents.

### 2.4. Sorption Procedures

The batch method (agitation technique) was used for carrying out the uranium sorption tests in closed bottles. Fixed volume of the solution (V, L), have a fixed concentration of the metal ions (C_0_, mmol L^−1^) at initial pH_0_ value (before sorption), this solution was mixed with a specific amount of the sorbent (m, g) in a specific time (t, hours) at room temperature (21 ± 1 °C).

For the uptake kinetics, the samples collected from this experiment at each specific time were filtrated using filter membrane (with specification 1.2 µm). The experimental conditions were carefully adjusted in the specific experiments. The standard condition is as follow; the sorbent with size below 10 µm were added to solution with initial metal concentration (C_0_) around 100 (±5) mg U L^−1^ (corresponding to 0.42 mmol U L^−1^); at initial pH value (pH_0_) 4; with sorbent dose (which described as amount of sorbent in a specific volume of the solution (SD; m/V)): 0.833 g L^−1^ and agitated for 24 h. The pH of the mixture wasn’t adjusted through the sorption processes but the pH_eq_ (equilibrium pH) was monitored after sorption experiment.

The sorption isotherms were used with different initial uranyl concentration that varied between 0.04 (±0.02) and 2.00 (±0.05) mmol U L^−1^. The models used for uptake kinetics and sorption isotherms are listed in Tables S1 and S2. Desorption experiments were investigated using 0.3 M HCl solution, it was tested from the sorbents collected after treatment in the kinetic experiments (uranium-loaded sorbent; around 0.650 g sorbent in each exp.). The condition used in the desorption procedure is as follow; the eluent volume 250 mL (SD: 2.52 (±0.02) g L^−1^) and the total time of desorption experiment is around 4 h. The recycling experiment was used with the same procedures in which, the water rinsing steps (approximately 30 (±5) mL) were performed between each sorption/desorption run.

The residual concentration (C_eq_, mmol U L^−1^) was analyzed using an inductively coupled plasma atomic emission spectrometer (ICP-AES) for synthetic and nature solutions. The uranium content in the diluted solution was measured using ICP-AES; Activa M; Horiba/France. The concentration of uranium ions from ore materials and leachate solution was analyzed using ammonium meta vanadate in oxidimetric titration method [47,48], while the measurements of the rare earth elements (REEs) were performed by colorimetric method using Arsenazo III at λ: 654 nm [49,50] through Shimadzu-160A; Shimadzu-Corporation, Kyoto-Japan. The sorption capacity for each element (q, mmol g^−1^) was measured by the mass balance equation: q = (C_0_ − C_eq_) × V/m.

### 2.5. Ore Specification

**Figure 1:**
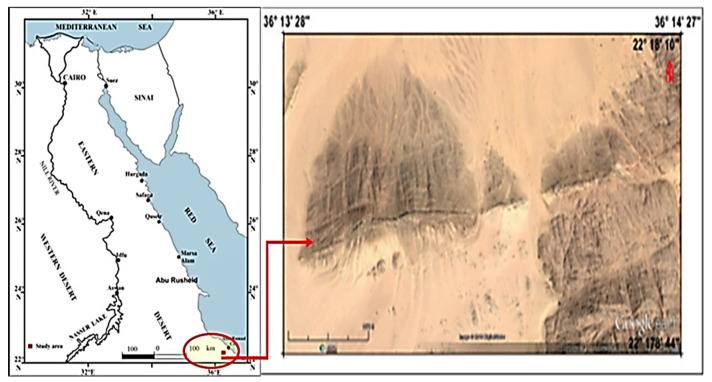
Google image, location map and geological map of El Sela area, South Eastern Desert, Egypt (after Nagar et al., 2016) [58].

Gabal (G) El-Sela area is considered as a signifying portion of late Precambrian early Paleozoic Pan Africa Orogeny. It involves two-mica granite as polymetallic intra-granitic vein-type U-deposits. It is known as the one of the most favorable areas in Egypt for hosting U-mineralization [51,52,53,54,55]. G El-Sela is sited in southern part of Egypt between latitudes 22°17′50″–22°18’06″ N and longitudes 36°13′36″–36°14′22″. It is in-between latitudes; (22°16′25″ N) and (22°18′40″ N), while the longitudes are described as (36°12′50″ E) and (36°16′30″ E). It is a topographically low, as well as moderate to slightly high, found as remarkably scattered isolated hills that are separated by vast sand sheets (Figure 1).

Many promising features to locate uranium mineralization were studied, particularly those related to the ENE-WSW shear zone [54,56]. Geology/mineralogy of G. El Sela two mica granite were premeditated by several researchers confirming prevalence of the primary and secondary uranium minerals [54,56,57]. The chemical composition of the collected sample that was used in this study was performed using XRF facilities and reported in Table 1. From chemical composition it was high concentration of U (1158 mg U kg^−1^), while some valuable elements were found in high concentration as REE (1330 mg U kg^−1^) and others in low concentration as V, Zr, Hf, Nb and Ta.

### 2.6. Mineralogical Characteristics of the Studied Sample

Mineralogical characteristics of the studied sample of the G. El Sela were firstly sieved to appropriate liberation size as determined microscopically (0.063–0.5 mm) and then separated into light and heavy fractions by using bromoform (sp. gr. 2.8 g cm^−3^). Different mineral grains were picked from the obtained heavy fractions and carefully investigated under binocular stereomicroscope. The separated grains were examined by SEM attached by EDX microanalysis unit for minerals identification. The results of EDX analysis reports of notable uranium minerals associated with non-radioactive elements. Accordingly, the studied ore material referred to the poly-mineral associations: autunite, uranophane and apatite in association with hematite, goethite and magnetite minerals.

### 2.7. Results of H_2_SO_4_ Agitation Leaching Process

From the foregoing study, it can be concluded that H_2_SO_4_ agitation leaching technique is more efficient for U leaching efficiency (89.7%) than other techniques (percolation and pelletization) from the studied G El-Sela at the optimum of the leaching conditions as 15% H_2_SO_4_ and adding 0.25 M NaCl with a 1/2 solid/liquid ratio at 2 h and 90 °C.

## 3. Results and Discussion

### 3.1. Sorbent Characterization

#### 3.1.1. Textural Properties

The functionalization process of the chitosan particles involved increasing in the SSA (specific surface area) through the adsorption/desorption of nitrogen isotherms. The SSA values were increased by functionalization from 5.75 m^2^ g^−1^ (for CH), to 13.7 m^2^ g^−1^ (for CH-S). This is due to the efficient reaction of the MBA and AMPS through crosslinking via C=C (lead to decreasing the size and high network) than that of MBA and chitosan (through C=C and NH_2_, respectively). According to this, the porous volume (volume of micropores) is increased as well from 0.0473 cm^3^ g^−1^ (for CH), to 0.0796 cm^3^ g^−1^ (for CH-S). The other criterion was observed through increasing the cumulative volumes (total volume) of pore from 0.051 to 0.059 cm^3^ g^−1^.

#### 3.1.2. Thermogravimetric Analysis

**Figure 2:**
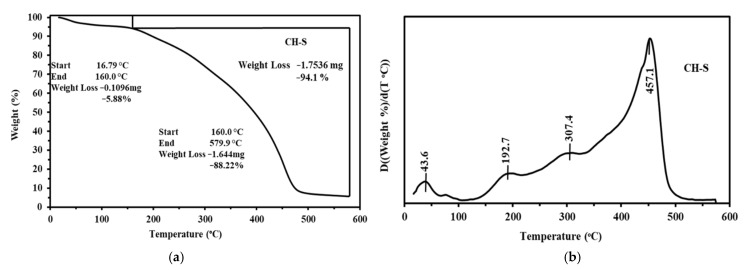
TGA (**a**) and DTG (**b**) for sulfonated chitosan sorbent (CH-S).

The thermal degradation and the derivative thermogram analyses (DTG) were performed for the functionalized chitosan sorbent (CH-S) as shown in Figure 2. It is characterized by two loss steps. The first stage is achieved at around 160 °C, this is attributed to the loss of the surface water (physically adsorbed) and inside the pores, the wight loss at this stage is 5.88%. The second stage of the loss is noticed between 160 °C and 579.9 °C, the additional loss was 88.22% and this was attributed to different modes for the polymer degradation, decomposition of the amine moieties from chitosan, degradation of sulfonic groups (which reports to be in the range of temp. 200–400 °C [59]), polymer network frame [60], and the char decomposition. It was concluded that the total weight loss of this polymer was close to 94.1%. Similar profiles were observed from Akköz et al. [61] through the use of sulfonated-agriculture waste. Several shoulders were found by DTG, the peaks at 43.6 and 192.7 °C for water loss either from the surface or inside the pores, while at 307.4 °C and 457.1 °C for depolymerization and degradation of the functional groups.

#### 3.1.3. FTIR Spectroscopy

**Figure 3:**
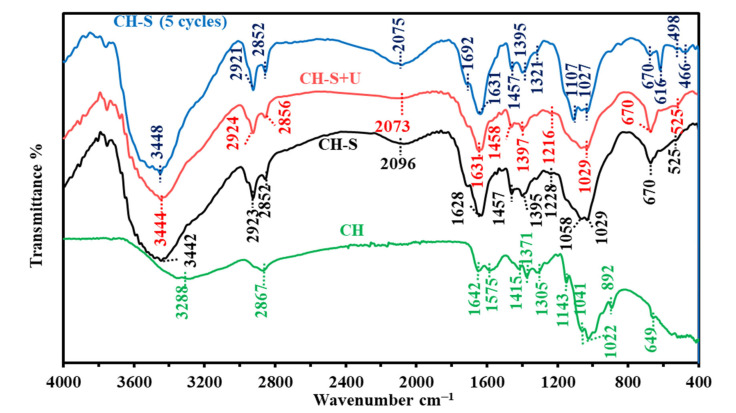
FTIR spectra of CH, CH-S, after loading and after 5 cycles of sorption desorption process (full wavenumber).

Figure 3 shows a comparison study of the crosslinked chitosan (before functionalization) and after functionalization process. The FTIR spectra present in this section represents with full wavenumber ranges, while Figure S1, shows the most important peaks for comparison the shifts and intensity during functionalization or loading and elution processes. The FTIR spectra were performed for sorbent before and after functionalization (CH and CH-S, respectively), after loading and after 5 cycles of sorption desorption for the CH-S. More specifically the -OH, -NH, and -SO_3_H groups are identified and reported in Table 2. As sulfonation was performed on chitosan a new series of peaks were identified associated with substantial changes, the C-S, O-S, and SO_3_H peaks were identified for the modified composite with increasing the intensity of OH and NH stretching bands (derived from the new substates and the sulfonic groups).

Increasing the intensity of the OH and NH bands as functionalization was performed was attributed to the new grafted groups (from the MBA and the sulphonic group) [62,63]. Disappearing of OH band at 649 cm^−1^ with the new broad band for C-O-S of sulfonic group was observed. Overlapping the N-H bands (which appeared at 1575 cm^−1^ in CH) with that of C=O for CH-S, verifying the changes occurred in the environment of this groups. There was an appearance of new broad band at 1096 cm^−1^ which is related to NCS group [64]. The other interested bands were reported in the Table 2. Most of these bands decreased in the intensity or shifts, especially for OH, NH, S-O, NCS, and C=O as uranyl ion adsorbed, which shared in the binding mechanism. After five cycles of sorption desorption processes, the spectra restored with full intensity of decreased peaks, confirming the high chemical stability and lack of changes in the chemical structure of the sorbent.

#### 3.1.4. Elemental Analysis

The elemental analysis was performed for each sorbent (CH and CH-S), which appeared in Table 3. As sulfonation was performed (by grafting of AMPS) on chitosan surface, with the MBA crosslinker, it followed an increase of N, O and S contents from 3.44, 26.408 and 0 mmol g^−1^ to 4.934, 26.952 and 0.683 mmol g^−1^, respectively.

**Figure 4:**
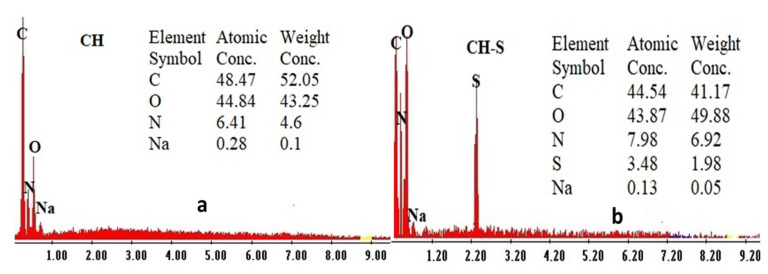
EDX analysis of CH (**a**) and CH-S (**b**).

Figure 4a,b, shows the semi-quantitative EDX analysis of the CH and after functionalization (CH-S). It shows a trace of Na element (from the sodium hydroxyl solution) in the final synthesis step. However, this analysis was parallel with the elemental analysis for increasing the N, and O as well as the appearance of S in the final sorbent, which is derived from AMPS and MBA.

#### 3.1.5. Surface Charge—pH_PZC_

**Figure 5:**
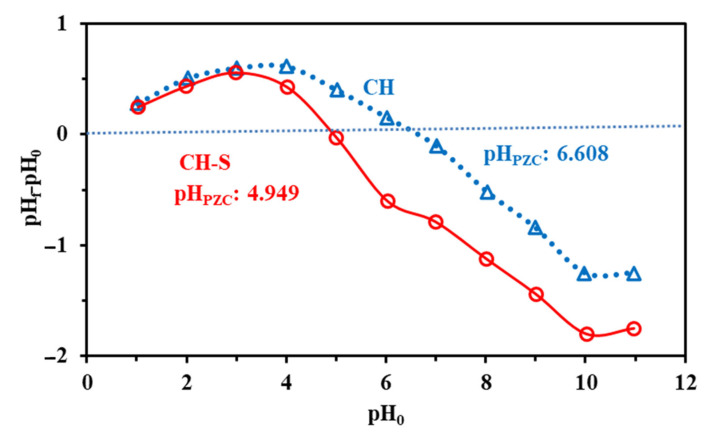
pH_pzc_ of the CH and CH-S sorbents.

Through applying the pH-drift method for determination of the pH_PZC_ values, Figure 5 shows the result of the pH_pzc_ value. It shows a shift based on acid base properties. The pH_PZC_ is strongly affected by functionalization of the sulfonic acid groups. In fact, the chitosan compound enhances the basic characters, which is derived from the amine and hydroxyl groups on the surface. By grafting the sulfonic acid moiety, the acid strength is noticeably changed and shifts to acidic part. The pH_PZC_ decreased from 6.6 to 4.9 for CH and CH-S, respectively. This reflects the effective functionalization of sulfonic groups on the chitosan behavior. On the other words, it means that, the surface of CH remains positively charged at a wide pH range than CH-S and this may cause a repulsion effect (especially at strong acidic pH) with positively charge metal ions. After pH 4.9 the CH-S surface becomes negative, this kind of repulsion is negligible even before this point. The sorbent is partially negatively charged, more specifically on the chelating atoms. Similar results (decreasing in the pH_pzc_ values) were obtained by Akkoz et al. through sulfonation of hawthorn kernel (as agriculture wastes) that decreased from 7 to 3.9 [61], while Urbano and Rivas [73] shows the pH_PZC_ values of 3.4 for the sulfonated groups of polyacrylamide and montmorillonite composites.

### 3.2. Sorption Studies

#### 3.2.1. pH Effect

**Figure 6:**
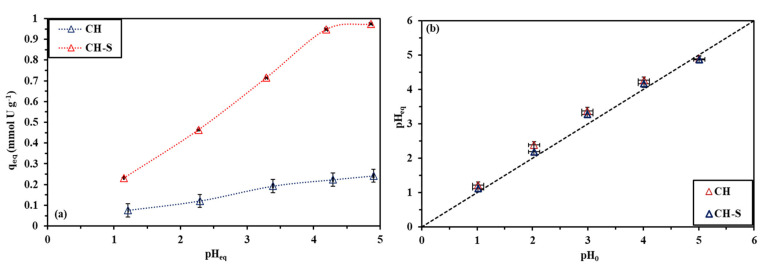
Effect of pH on uranyl sorption (**a**) and pH variation of the three experiments (**b**).

Figure 6a compares the loading capacities of both sorbents (before and after modification) toward U (VI) ions. Regardless of the type of sorbent, the sorption capacity of U (VI) was progressively increased as pH raised from 1 to 5 before becoming stabilized at pH around 4. It was increased from 0.08 to 0.23 ± 0.02 mmol U g^−1^ for CH sorbent, and from 0.22 to 0.95 ± 0.05 mmol U g^−1^ for CH-S. From these data, it was shown that as functionalization was performed, the loading capacity enhanced by more than 4 times. Increasing the pH caused partial deprotonation of the functional groups and the electrons on the N, O and S are ready for binding through chelating properties. While repulsion between positively charged uranyl ions and protonated groups at acidic pH values exists, on the other side the sorption properties were improved by increasing pH through decreasing the repulsion effects. The three repeated experiments show the successes in the reproducibility properties of the sorbent. Figure 6b shows the pH deviation of the three experiments, it shows deviation of about 0.3 values. From Figure S2, the uranium was found in different species depending on the pH values. The most interesting points are those that lay below pH 4 and 5 for the maximum sorption, before the precipitation point (>5.2) and the existing of hydrolyzed species. The uranyl oxide (UO_2_^2+^) is the mainly sorbed species that are found in the solution (for our case/C_0_, 100 mg L^−1^, uranyl nitrate as uranium source, at pH 4), which can bound with the deprotonated (or partially deprotonated) functional groups as NH, OH and SO_3_H.

#### 3.2.2. Uptake Kinetics

**Figure 7:**
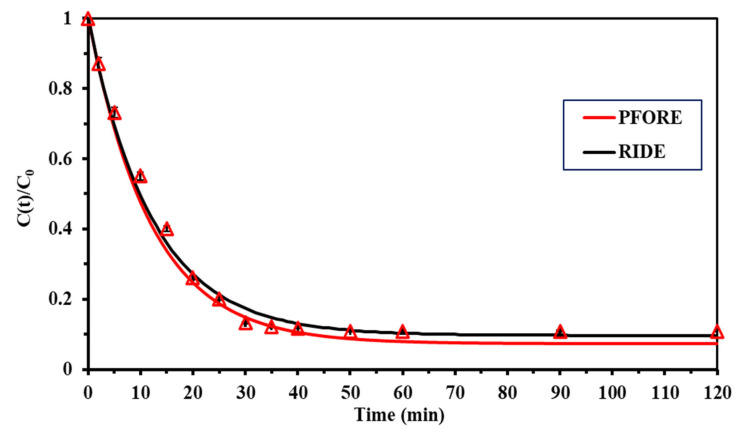
Uptake Kinetic models PFORE and RIDE that fitted the experimental data.

Under the selected experimental conditions (for the pH, sorbent dose and uranium concentration in the solution), the sorption equilibrium is reached during the first 40 min, while around 90% of the total sorption was attained during 25 min as shown in Figure 7 and Figure S3. The superimposition of the three experiments (small in deviation error), which emphasizes the reproducibility. The large pores character (see BET surface area) of the sorbent may explain the fast sorption kinetics and the fast mass transfer, which is controlled through the resistances to film diffusion, as well as the intraparticle diffusion properties, so the resistance to intraparticle diffusion equation (RIDE) cannot be neglected for the kinetic models. Moreover, the proper sorption rate, which is modeled by pseudo-first order (PFORE); it is described as physical sorption, or pseudo-second order rate equation, PSORE, by correspond the chemical sorption.

Hubbe et al. [74] concluded that the PSORE in a lot of cases was inferred with the control of mass transfer through resistance to intraparticle diffusion. Simonin [75] was declared the critical impacts of selection of experimental condition, which was responsible for the quality of the models used (i.e., PFORE and PSORE).

Table S1 summarizes the different equations which used in describing of the kinetic profiles. Table 4 reports the experimental values and the parameters for uranium sorption. The values of (R^2^) of the determination coefficient and the comparisons of both experimental and calculated of the sorption parameters (C(t)_fitted_/C_0_ vs. C(t)_exp_/C_0_) are demonstrated that the PFORE and RIDE are more appropriate for kinetic profile modeling. This is also confirmed by the values of AIC, which indicating the lowest (negative) values obtained by these two models.

#### 3.2.3. Sorption Isotherms

**Figure 8:**
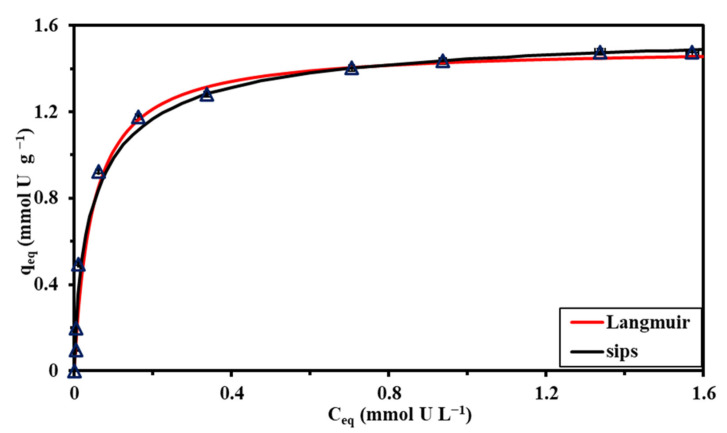
Sorption isotherms models for the experimental data of Langmuir and SIPS equations.

Figure 8 shows the plotted of sorption isotherms (with error bars) for U(VI) sorption from the mono component solution at pH_in_ 4 ± 0.1 (pH_eq_: 4.1 ± 0.1) using CH-S sorbent. Figure S4 reports the Freundlich and Temkin models for fitting the sorption isotherms of U sorption. It was shown that the sorption capacities are progressively increased with concentration before tends to the saturation plateau (which seems to be high for U(VI) 1.54 mmol U g^−1^).

The sorption of uranium is controlled by the pH of the solution as well as the uranium species (mainly UO_2_^2+^). The fitted equation for the isotherm profiles is the Langmuir equation. This equation is considered as a good fitting model of the experimental data over the entire range of the uranyl concentrations. The Freundlich equation (fails for fitting the sorption experiments either in the initial or saturation sections) is a power-like equation. Sips equation (has a good fit in the whole sections), which includes an adjustable parameter (n). From the data collected from Table 5 concerns with the R^2^, variations coefficient of the affinity and AIC values (including the triplicate experiments), the preferred models for fitting the data in Figure 8 is the Langmuir and the Sips equations. The sorption capacity at saturation of the monolayer (i.e., q_m,L_) slightly overestimates the experimental values.

Table 6 reports the comparison of U(VI) sorption of some alternative sorbents. It is hard to establish such a comparison due to different functional groups (the bases used) and the detected solution (the source of uranium bearing solution, initial concentration of the uranium ions in either nature liquids or synthetic solution and the uranium salt used in the synthetic solution) but try to make select the matches condition for our work. The sorption capacity is relatively with high values (1.5 mmol U g^−1^). Some sorbents were used at pH 6, with high loading capacity (4.48 mmol g^−1^) using functionalized silica with diethylenetriamine tethered, but there is a risk in our cases of using such condition to avoid precipitation [76]. The other functionalized of mesoporous silica with Phosphonate groups [77] but with lower level of affinity and capacity. Others derived from marine fungus with capacity (about 1.56 mmol U g^−1^) but exhibited a lower affinity coefficient (≈0.38 L mmol^−1^) [78]. The sorbent represents a promising tool for extraction of uranium with respect the kinetic uptake, the capacity, and the affinity coefficients.

#### 3.2.4. Sorption Mechanism

**Figure 9:**
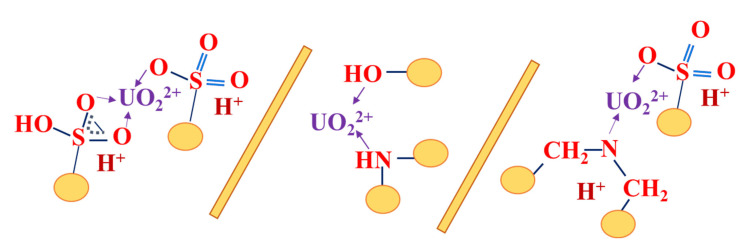
Suggest binding mechanism of functionalized sorbent toward U(VI) ions.

The collected information from the pH_PZC_ (global charge of the functional groups) and the sorption behavior during different pH values (depending on the speciation of uranyl ions), FTIR analysis (shifts and change the peaks resolution, which used in the interaction models; mainly for OH, SO_3_H and NH), and the data of the EDX analyses (increasing the ratio of some components as S element during synthesis and sorption of uranyl ions, which appeared clearly). It is possible to conclude a different interaction mode of the reactive functional groups on CH-S sorbent and the uranyl ions. The interaction expected to perform through protons on either sulfonic acid, and hydroxyl groups beside the free electrons available on the amine groups, this interaction be mixed of ion exchange and chelating modes, especially that the sorbent is completely negative, and the optimism sorption pH as shown in Figure 9.

#### 3.2.5. Metal Desorption and Recycling Properties

**Figure 10:**
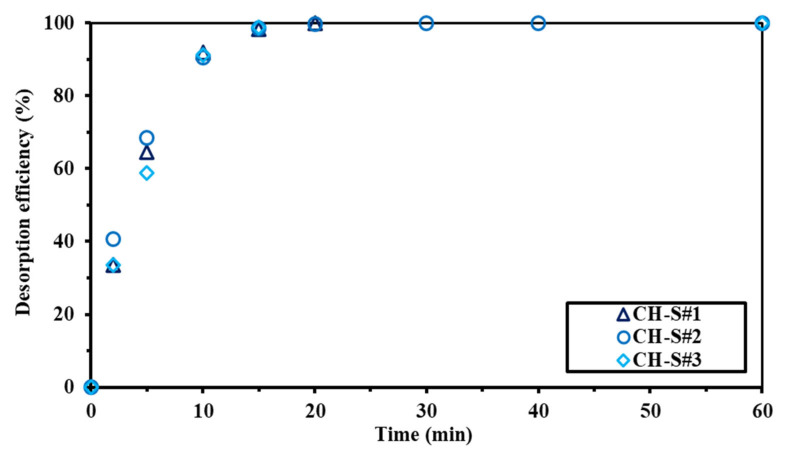
Desorption kinetics of uranium on CH-S sorbent.

Desorption of adsorbed uranyl ions and recycling of the CH-S sorbent is a key of challenge in valorization and in term of metal recovery. This is the important step in evaluating of the competitiveness process. From FTIR spectra, it was shown the chemical stability of sorbent during recycling process and restoring the original peaks of the sorbent. The composite material produced from the kinetic experiments applied for desorption using 0.3 M HCl solution, which shows fast kinetics. The maximum time required for complete desorption is around 20 min as shown in Figure 10.

From Table 7 shows stability of sorption and desorption recycling which shows a remarkable stability in both sorption (with limited decreasing; around 3% after five cycles) and the desorption efficiency which remains stable (higher than 99.5%). This means that, the prepared composite is shown a remarkably stable of reuses.

#### 3.2.6. Results of the Uranium Extraction

Results of the uranium extraction applying the optimum leaching conditions upon 200 g of El Sela highly altered granitic rocks. Agitation leaching technique using sulfuric are more efficient than other techniques (percolation and pelletization). Treatment of the collected ore is sampled with 15% H_2_SO_4_ in the presence of 0.25 M NaCl with a 1/2 of solid/liquid ratio for 2 h at 90 °C, which yields a solution with uranium leaching efficiency of 89.7%. The produced leaching liquor from 200 g ore sample is about 0.6 L of sulfate solution. The produced solution contains 386 mg U L^−1^ with pH 0.3. The most interesting metal ions concentration is given in Table 8.

**Figure 11:**
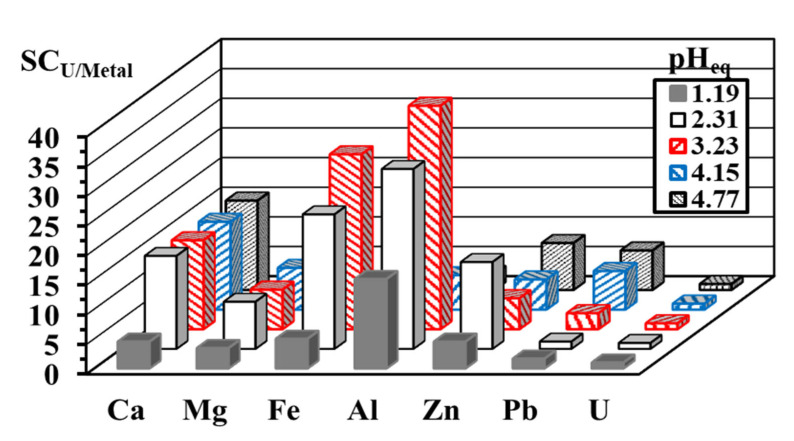
Selectivity studies of uranium sorption from acidic ore leachate.

The sorption was performed in batch method at different pH values 1 to 5. As pH increased, the sorption efficiency also increased. The capacity of uranium increased from 0.03, 0.156, 0.20, 0.224 and 0.253 mmol U g^−1^ from pH_eq_ 1.2, 2.3, 3.2, 4.1 to 4.8, respectively. The selectivity coefficient (SC) was increased to pH_eq_ 3.2 then decreased to pH_eq_ 4.8. The SC_U/Metal_ was shown in Figure 11 at the optimum pH_eq_ 3.2, which is around 14.99, 6.67, 29.49, 37.68, 5.22 and 2.70 for Ca, Mg, Fe, Al, Zn, and Pb, respectively. This indicates the efficient uses of the modified chitosan for uranium removal from low concentrate solution. This can be used for the heavy metal removal of Fe, Zn, and Pb from aquatic medium, which appear to have highly efficient sorption toward these ions.

## 4. Conclusions

Successive synthesis of novel sorbent based on chitosan particles for high performance uranium recovery was performed through functionalization of sulfonic groups (source; AMPS) on the chitosan surface via redox polymerization. The produced hydrogel was characterized by FTIR spectroscopy, TGA analysis, textural properties (BET surface area) through nitrogen adsorption method, pH_PZC_ (by pH-drift method), SEM, and SEM-EDX analyses. The most favorable pH is around 4 (prior to precipitation limit), the sorption kinetics is relatively fast; it is around 25 min is sufficient for total sorption, and 15 min for the total desorption with more than 99.9% of desorption efficiency. Using 0.3 M HCl solution is efficient eluent. The PFORE and RIDE are the most fitting model for kinetic uptake than the PSORE, while Langmuir and Sips equations for sorption isotherms. The sorbent was applied for uranium recovery from leachate waste solution and is considered as a promising tool for the recovery of uranium from acid leaching in polymetallic solution.

## Figures and Tables

**Table 1:** XRF analysis of the study G. El Sela raw materials.

Major Oxides (%)	Wt. (%)	Trace Elements	ppm
SiO_2_	76.59	U	1158
Al_2_O_3_	8.6	REE	1330
TiO_2_	1.03	Th	16
Fe_2_O_3_^total^	3.76	Mn	36
CaO	1.22	V	48
MgO	0.46	Zn	79
Na_2_O	0.42	Pb	139
K_2_O	1.27	Zr	177
P_2_O_5_	0.51	Hf	5.4
L.O.I	5.7	Nb	58
Total	99.56	Ta	2.7

**Table 2:** Assignments peaks in FTIR spectra for CH and CH-S (before and after U(VI) sorption, and after five cycles of sorption and desorption).

Assignment	CH	CH-S	CH-S + U	CH-S (5th Cycle)	Ref.
O-H and N-H (stretching)	3288	3442	3444	3448	[65,66]
C-H (stretching)	2867	2923, 2852	2924, 2856	2921, 2852	[66,67]
-NCS group		2096	2073	2075	[68]
C=O (stretching) and NH of amide	1642, 1575	1628	1631	1692, 1631	[66,69]
-CH_2_ (bending) and C-N (stretching)	1415	1457	1458	1457	[66,69]
CH_3_ (symmetric deformation)	1371, 1305	1395	1397	1395, 1321	[66,67,69]
-N-C- (stretching) and sulfonamide group		1228	1216	1107	[68]
C-O-C (stretching)	1143				
C-O (skeletal stretching) and C-H out-of-plane (bending)	1041, 1022	1058, 1029	1029	1027	[65,66]
C-O- of epoxy ring	892				
C-O-S (stretching) and -(CH_2_)_n_- rocking	649	670	670	670, 616	[68]
O-H out of plane (bending), C-S and/or C-O-S (stretching).		525	525	498	[70,71,72]
Polysulfides(S-S stretching)				466	our

**Table 3:** Elemental analysis of CH and CH-S sorbents.

Sorbent	C	N	H	O	S
CH (%)	45.94	4.83	6.98	42.25	0
CH (mmol g^−1^)	38.248	3.449	69.253	26.408	0
CH-S (%)	41.56	6.91	6.22	43.12	2.19
CH-S (mmol g^−1^)	34.602	4.934	61.712	26.952	0.683

**Table 4:** The parameters of the three models for U(VI) uptake kinetics for the three experiments.

Model	Parameter	1	2	3
Exp.	q_eq_ (mmol U g^−1^)	0.968	0.966	0.959
PFORE	q_eq,1_ (mmol U g^−1^)	0.978	0.974	0.966
	k_1_ × 10^2^ (min^−1^)	3.27	3.71	3.28
	R^2^	0.995	0.979	0.989
	AIC	−136.6	−146.8	−144.9
PSORE	q_eq,2_ (mmol U g^−1^)	0.802	0.796	0.817
	k_2_ × 10^2^ (L mmol^−1^ min^−1^)	11.7	9.87	10.68
	R^2^	0.797	0.815	0.856
	AIC	−48.3	−39.7	−49.3
RIDE	D_e_ × 10^8^ (m^2^ min^−1^)	2.19	1.97	2.05
	R^2^	0.992	0.989	0.973
	AIC *	−137	−143.3	−144.5

* Where AIC means Akaike Information Criterion and discusses in Table S2.

**Table 5:** Modeling of U(VI) sorption isotherms.

Model	Parameter		1	2	3
Experimental	q_m,exp._	mmol U g^−1^	1.543	1.477	1.43
Langmuir	q_m,L_	mmol U g^−1^	1.572	1.483	1.451
	b_L_	L mmol^−1^	1.32	1.29	1.31
	R^2^	-	0.993	0.984	0.979
	AIC	-	−144	−147	−169
Freundlich	k_F_	mmol^1−1/n^ g^−1^ L^1/n^	0.85	0.79	0.68
	n_F_	-	2.1	2.51	35
	R^2^	-	0.894	0.911	0.928
	AIC	-	−74.3	−69.3	−58.4
Sips	q_m,S_	mmol U g^−1^	1.59	1.481	1.447
	b_S_	L mmol^−1^	1.17	1.08	1.14
	n_S_	-	1.16	1.23	1.27
	R^2^	-	0.993	0.989	0.981
	AIC	-	−152	−176	−185
Temkin	A_T_ × 10^−3^	L mmol^−1^	22.56	30.2	28.1
	b_T_	kJ mol^−1^	17.4	16.4	15.3
	R^2^	-	0.786	0.769	0.832
	AIC	-	−36	−46	−25

**Table 6:** U(VI) sorption properties of alternative sorbents.

Sorbent	pH	t_eq_	q_m,exp_	q_m,L_	b_L_	Ref.
Functionalized of the activated carbon	5	140	-	0.808	81.4	[79]
Phosphonated mesoporous silica	4	10	1.48	1.15	12.6	[77]
Duolite (ES-467)	3	90	-	0.326	10.9	[80]
Functionalized magnetic with amino and phosphine oxide composite	0.5	180	0.727	0.825	5.47	[81]
Functionalized polyethylene non-woven fabrics	4	720	0.084	0.087	119	[82]
Cyanobacterium	5	60	0.708	0.799	28.6	[83]
Manganese oxide functionalized with amine	4	20	-	0.416	19.0	[84]
Water hyacinth-biochar	6	720	0.571	0.663	407	[85]
201X8 ion exchanger	1.57	120	-	0.282	5.71	[86]
Trimesoyl chloride-melamine-palygorskite composite	6	75	-	1.16	0.95	[87]
Carboxylated chitosan nanotubes aerogels	5	150	1.07	1.29	23.6	[88]
activated carbon-Nano-HAP-alginate	6	480	0.042	0.078	0.95	[89]
Urea-formaldehyde resin	6	180	0.412	0.417	381	[90]
Chitosan/Amidoxime PAN/GO	6	120	1.04	1.24	169	[91]
Carboxymethyl konjac-glucomannan-gellan gum	6	720	0.411	0.428	65.0	[92]
*Yarrowia lipolytica*-alginate beads	7.5	90	-	0.102	2.86	[93]
Amidoxime-marine fungus	5	120	1.56	1.56	0.38	[78]
TiO_2_-alginate	5	1440	0.105	0.132	-	[94]
Gum-ghatti polyacrylamide	6	240	-	1.54	29.8	[95]
Resin of carminic acid	5	120	0.807	0.808	36.3	[96]
Functionalized picolylamine resin	5.3	120	2.10	2.31	164	[97]
Chitosan film	5	600	0.735	0.827	223	[98]
Colloid montmorillonite	6	60	-	0.076	187	[99]
Impregnated bentonite	4	15	-	0.268	40.0	[100]
Dowex 50W X8/Alizarin Red-S	3	30	-	0.512	141	[101]
Acrylic Membrane/Algal fiber	3	60	0.260	0.268	9.05	[102]
Urea/SiO_2_	4	90	1.14	1.17	21.3	[18]
Thiourea/SiO_2_	4	90	1.02	1.16	5.98	[18]
Silica gel with β-cyclodextrin/	4.5	60	-	0.070	50.3	[103]
silica Functionalized MCM-41	5	40	1.89	2.00	850	[104]
PGG@C	4	60	1.28	1.32	47.1	[105]
PGG@MC	4	60	1.15	1.22	22.0	[105]
CH-S	4	30	1.53	1.55	1.3	This work

Units: t_eq_: min; q_m_: mmol U g^−1^; b_L_: L mmol^−1^.

**Table 7:** Sorption and desorption recycling for the sorbent toward uranium (VI) ions.

	Sorption		Desorption	
Cycles	Removal Effeciency (%)	S.D. (Re. %)	Desorption (%)	S.D. (De %)
1	97.6857	0.73187	99.846	0.22026
2	97.003	0.72855	99.893	0.14502
3	96.4471	0.79283	99.989	0.17285
4	96.1154	0.71282	99.672	0.12039
5	95.6361	0. 7312	99.864	0.17931

**Table 8:** Chemical composition of the prepared leach liquor at (pH = 0.3).

Constituants	Conc. (mgL^−1^)	Constituants	Conc. (mgL^−1^)
U	386	Al_2_O_3_	2540
REE	448	Pb	20
Fe	2700	Zr	29

## Data Availability

Data available from authors.

## Supplementary Materials

The following supporting information can be downloaded at: https://www.mdpi.com/article/10.3390/toxics10090490/s1, Table S1: Reminder on equations used for modeling uptake kinetics [106,107]; Table S2: Reminder on equations used for modeling sorption isotherms [108,109,110,111,112,113]; Figure S1: FTIR spectra of most interested vibrational bands for CH, CH-S, after loading and after 5 cycles of sorption desorption process; Figure S2: Uranyl species with different pH values; Figure S3: The PSORE of the uptake kinetics; Figure S4: The Freundlich and Tamkin models for application to fit the sorption isotherms.
