# Supplementary material for: Ecofriendly Composite as a Promising Material for Highly-Performance Uranium Recovery from Different Solutions
Source: Toxics. 2022 Aug 24;10(9):490. doi: 10.3390/toxics10090490 (PMC9502357; doi:10.3390/toxics10090490)
Supplement: Supplementary file 1 [file toxics-10-00490-s001.zip › toxics-1862407-supplementary.pdf]

toxics

Article

Ecofriendly Composite as a Promising Material for Highly-Performance Uranium Recovery from Different Solutions

Mohammed F. Hamza,[1,2]* Hanaa A. Abu Khoziem,[2] Mahmoud S. Khalafalla,[2] Walid M. Abdellah[2], Doaa I. Zaki[2], Khalid Althumayri[3], Yuezhou WEI,[1,4]*

[1] School of Nuclear Science and Technology, University of South China, Heng Yang 421001, China
[2] Nuclear Materials Authority, POB 530, El-Maadi, Cairo, Egypt
[3] Department of chemistry, College of science, Taibah University, 30002 Al-Madinah Al-Munawarah, Saudi Arabia
[4] School of Nuclear Science and Engineering, Shanghai Jiao Tong University, Shanghai, China
* M. F. Hamza m_fouda21@hotmail.com; +2-01116681228, Y. Wei: yzwei@sjtu.edu.cn +86-771-3224990

Citation: Hamza, M.F.; Abu Khoziem, H.A.; Khalafalla, M.S.; Abdellah, W.M.; Zaki, D.I.; Althumayri, K.; Wei, Y. Ecofriendly Composite as a Promising Material for Highly-Performance Uranium Recovery from Different Solutions. *Toxics* **2022**, *10*, 490. https://doi.org/10.3390/toxics10090490

Academic Editor: Xiaojun Luo

Received: 28 July 2022
Accepted: 18 August 2022
Published: 24 August 2022

Publisher's Note: MDPI stays neutral with regard to jurisdictional claims in published maps and institutional affiliations.

Copyright: © 2022 by the authors. Licensee MDPI, Basel, Switzerland. This article is an open access article distributed under the terms and conditions of the Creative Commons Attribution (CC BY) license (https://creativecommons.org/licenses/by/4.0/).

Table S1. Reminder on equations used for modeling uptake kinetics [1,2].

Model	Equation	Parameters	Ref.
PFORE	$q(t) = q_{eq,1}(1 - e^{-k_1 t})$	$q_{eq,1}$ (mmol g^{-1}): sorption capacity at equilibrium<br>k_1 (min^{-1}): apparent rate constant of PFORE	[1]
PSORE	$q(t) = \frac{q_{eq,2}^2 k_2 t}{1 + k_2 q_{eq,2} t}$	$q_{eq,2}$ (mmol g^{-1}): sorption capacity at equilibrium<br>k_2 (g $mmol^{-1}$ min^{-1}): apparent rate constant of PSORE	[1]
RIDE	$\frac{q(t)}{q_{eq}} = 1 - \sum_{n=1}^{\infty} \frac{6\alpha(\alpha+1)\exp\left(\frac{-D_e q_n^2}{r^2} t\right)}{9 + 9\alpha + q_n^2 \alpha^2}$<br>With q_n being the non-zero roots of<br>$\tan q_n = \frac{3\, q_n}{3 + \alpha q_n^2}$ and $\frac{m\, q}{V\, C_0} = \frac{1}{1+\alpha}$	D_e (m^2 min^{-1}) : Effective diffusivity coefficient	[2]

(m (g): mass of sorbent; V (L): volume of solution; C_0 (mmol L^{-1}): initial concentration of the solution).

Table S2. Reminder on equations used for modeling sorption isotherms.

Model	Equation	Parameters	Ref.
Langmuir	$q_{eq} = \frac{q_{m,L} C_{eq}}{1 + b_L C_{eq}}$	$q_{m,L}$ (mmol g^{-1}): Sorption capacity at saturation of monolayer<br>b_L (L $mmol^{-1}$): Affinity coefficient	[3]
Freundlich	$q_{eq} = k_F C_{eq}^{1/n_F}$	k_F (mmol g^{-1})/(mmol L^{-1})^{n_F} and n_F: empirical parameters of Freundlich equation	[4]
Sips	$q_{eq} = \frac{q_{m,S} b_S C_{eq}^{1/n_S}}{1 + b_S C_{eq}^{1/n_S}}$	$q_{m,L}$ (mmol g^{-1}), b_S (mmol L^{-1})^{n_S}, and n_S: empirical parameters of Sips equation (based on Langmuir and Freundlich equations)	[5]
Temkin	$q_{eq} = \frac{RT}{b_T} \ln\left(A_T\, C_{eq}\right)$	A_T (L $mmol^{-1}$): equilibrium binding capacity;<br>b_T: Temkin constant related to sorption heat (J kg^{-1} mol^{-2})	[6]
D-R*	$q_{eq} = q_{m,DR} exp\left\{-\beta_{DR}\left[RT\, ln\left(1 + \frac{1}{C_{eq}}\right)\right]^2\right\}$	$q_{m,DR}$ (mmol g^{-1}): maximum adsorption capacity;<br>β_{DR} (mol^2 kJ^{-2}): constant associated with adsorption energy.<br>$E_{DR} = \frac{1}{\sqrt{2\beta_{DR}}}$: mean free energy of sorption (kJ mol^{-1})	[7]

, herein C_{eq} must be expressed in molar unit for respecting the dimensionless term ($1/C_{eq} = C^*/Ceq$, where C is the arbitrary concentration

Akaike Information Criterion, AIC [8]:

$$AIC = N \ln\left(\frac{\sum_{i=0}^{N}\left(y_{i,exp.} - y_{i,model}\right)^2}{N}\right) + 2N_p + \frac{2N_p\left(N_p + 1\right)}{N - N_p - 1}$$

Where N is the number of experimental points, N_p the number of model parameters, $y_{i,exp.}$ and $y_{i,model}$ the experimental and calculated values of the tested variable.

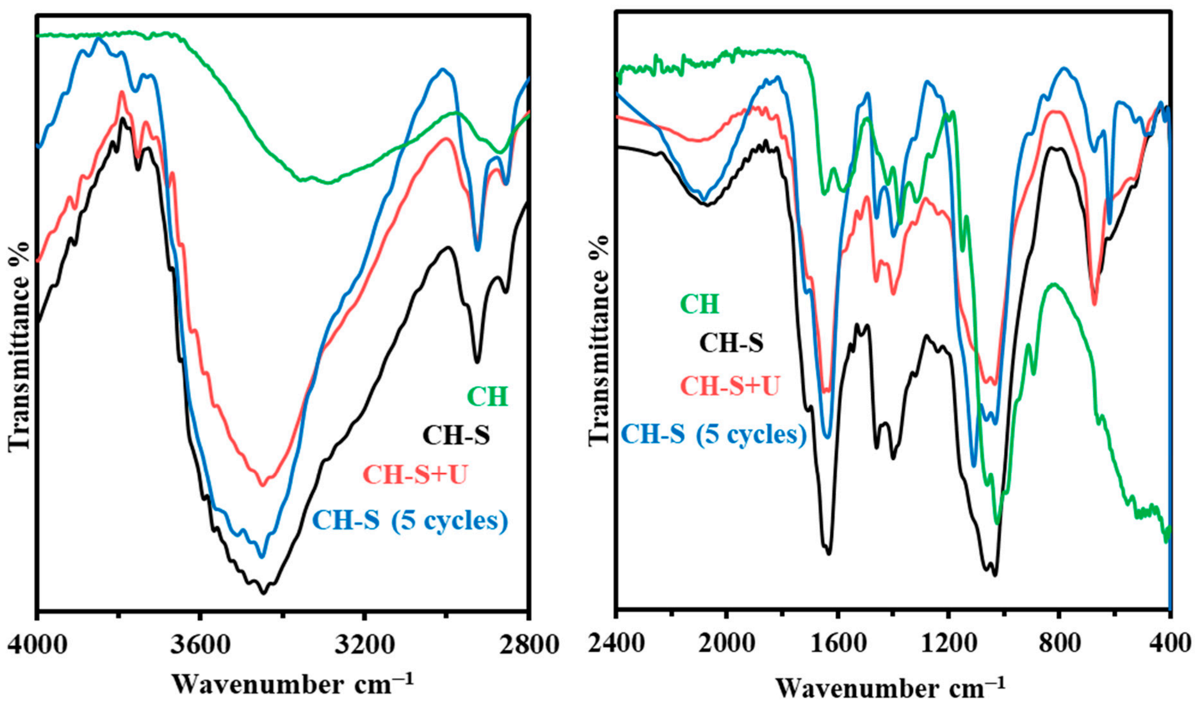

Figure S1. FTIR spectra of most interested vibrational bands for CH, CH-S, after loading and after 5 cycles of sorption desorption process.

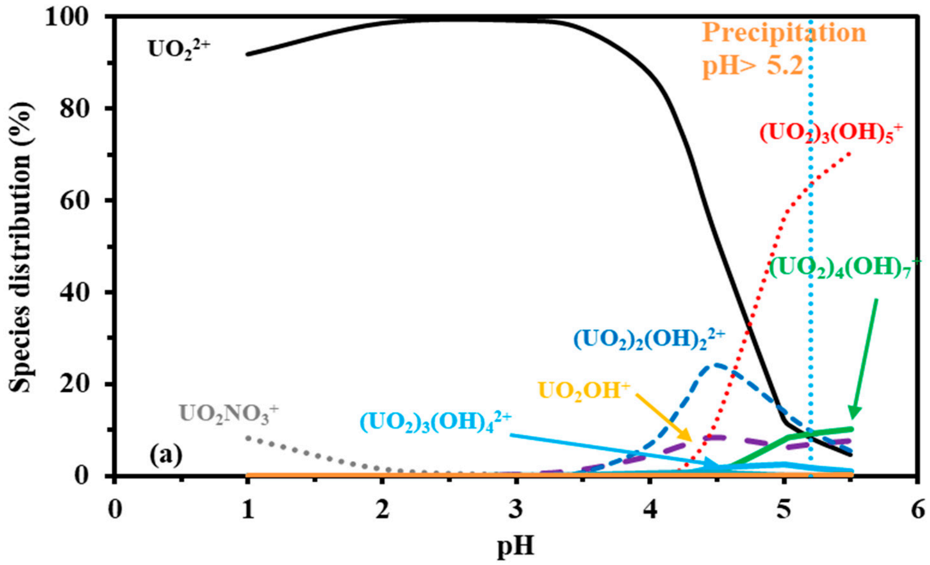

Figure S2. Uranyl species with different pH values.

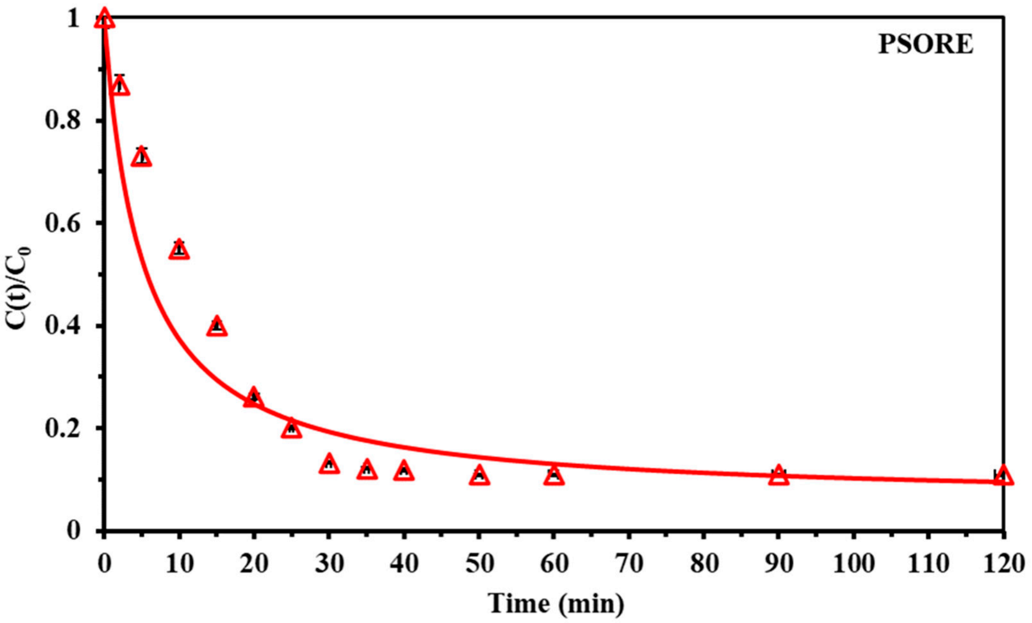

Figure S3. The PSORE of the uptake kinetics.

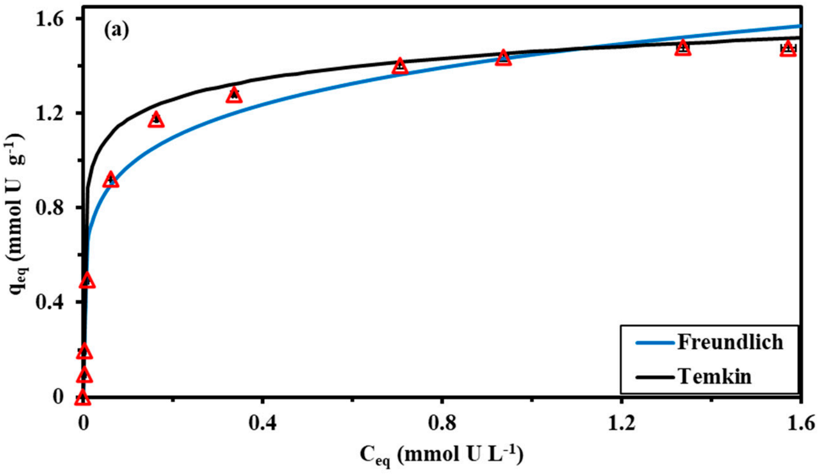

Figure S4. The Freundlich and Tamkin models for application to fit the sorption isotherms.

References

1. Ho, Y.S.; McKay, G. Pseudo-second order model for sorption processes. *Process Biochem.* **1999**, *34*, 451–465. https://doi.org/10.1016/S0032-9592(98)00112-5.
2. Crank, J. *The Mathematics of Diffusion*, 2nd ed.; Oxford University Press: Oxford, UK, 1975; p. 414.
3. Langmuir, I. The adsorption of gases on plane surfaces of glass, mica and platinum. *J. Amer. Chem. Soc.* **1918**, *40*, 1361–1402.
4. Freundlich, H.M.F. Uber die adsorption in lasungen. *Z. Phys. Chem.* **1906**, *57*, 385–470.
5. Tien, C. *Adsorption Calculations and Modeling*; Butterworth-Heinemann: Newton, MA, USA, 1994; p. 243.
6. Kegl, T.; Kosak, A.; Lobnik, A.; Novak, Z.; Kralj, A.K.; Ban, I. Adsorption of rare earth metals from wastewater by nanomaterials: A review. *J. Hazard. Mater.* **2020**, *386*, 121632. https://doi.org/10.1016/j.jhazmat.2019.121632.
7. Puccia, V.; Avena, M.J. On the use of the Dubinin-Radushkevich equation to distinguish between physical and chemical adsorption at the solid-water interface. *Colloid Interface Sci. Commun.* **2021**, *41*, 100376.
8. Falyouna, O.; Eljamal, O.; Maamoun, I.; Tahara, A.; Sugihara, Y. Magnetic zeolite synthesis for efficient removal of cesium in a lab-scale continuous treatment system. *J. Colloid Interface Sci.* **2020**, *571*, 66–79. https://doi.org/10.1016/j.jcis.2020.03.028.
